# Digital Fellowships: Inspiring use of contemporary technologies in applied healthcare

**DOI:** 10.1038/s41746-023-00922-8

**Published:** 2023-09-26

**Authors:** Thomas Salisbury, Alexander T. Deng, Emily Burch, Alan Godfrey

**Affiliations:** 1 James Cook Hospital, Middlesbrough, UK; 2 Guy’s and St Thomas’ NHS Foundation Trust, London, UK; 3 Barnet, Enfield and Haringey Mental Health Trust, London, UK; 4 Department of Computer and Information Sciences, Northumbria University, Newcastle, UK

**Keywords:** Education

## Abstract

The adoption of digital technologies in healthcare, accelerated by the COVID-19 pandemic, requires a well-prepared workforce capable of implementing those technologies. Here, we examine the role and impact of digital fellowships in facilitating digital transformation in healthcare systems. Digital fellowships are structured educational programmes designed to equip healthcare professionals with advanced digital skills. Focusing on UK-based initiatives like the Topol Digital Fellowship and the Fellowship in Clinical AI, we explore their efforts to prepare healthcare leaders for digital and AI adoption. Each fellowship programme provides participants with hands-on experience in digital healthcare projects and fosters interdisciplinary collaboration and post-fellowship support. We discuss how these fellowships contribute to staff retention by diversifying professional experiences and opportunities. We call for increased collaborations between universities, industry, and professional bodies to integrate lessons from digital fellowships into relevant curricula, acknowledging that digital fellowships are just one piece of the puzzle in bridging the digital skills gap in the healthcare workforce.

## Introduction

The COVID-19 pandemic jolted the healthcare sector into accelerating the adoption of contemporary digital technologies to address urgent unmet needs. Adoption could better enable remote assessment of people at home, or to expedite the flow of patients within their care pathway. Those technologies include the use of telemedicine, mobile health, artificial intelligence, and electronic health records^1^. The arising paradigm shift in use of digital technologies has the potential to transform and empower both patients and clinicians by improving efficiency and patient outcomes. However, a key challenge in implementing those transformations lies in the need for a well-prepared workforce capable of seamlessly integrating and optimising digital tools. How can a workforce be prepared to proactively lead in digital transformation, instead of reacting to external crises?

A previous article in this journal outlined the essential components for establishing a strategic, mission-driven approach to healthcare and highlighted the importance of nurturing in-house expertise^2^. In the UK the recently published NHS Long Term Workforce Plan^3^ attempts to outline strategies for workforce training, retention, and reform. The plan puts digital at the forefront and it illustrates some of the ways in which the digital workforce can be trained through organisations such as The NHS Digital Academy^4^, which offers programmes like the Digital Leadership Programme and Digital Fellowships, as well as providing resources like the AI and Digital Healthcare Technologies Capability Framework^5^. Here, we highlight the role of digital fellowships as catalysts for future-oriented, resilient, and adaptable workforce in an increasingly digital world.

### The emergence of digital fellowships

A *fellowship* denotes a structured educational programme designed to cultivate advanced knowledge and skills within a specific field or discipline^6^. In contrast to postgraduate degrees such as master’s programmes (which generally provide a comprehensive and theoretical foundation in a subject), fellowships prioritise specialised, real-world, and often experiential learning tailored to address immediate challenges and cutting-edge developments within a niche domain. Digital fellowships within healthcare are undertaken alongside professional training or after qualification and are designed to equip fellows with specialised capabilities beyond their standard curricula. Digital fellowships typically last 12 months. While they demand dedication, they’re often flexibly structured and provide fellows with the gift of time to focus on roles outside of their day-to-day activities. Many fellowship posts are undertaken part time, in tandem with existing roles, though others involve a full-year secondment. Lessons from both industry and government show digital transformation is most effective when it is a collaborative endeavour^7^, from the core of an organisation. As the NHS faces growing pressure to deliver efficient and patient-focused care, digital fellowships have emerged as a means of cultivating such agents of change from within.

Fellows, often dissatisfied with the prevailing technological or cultural trajectory, can act as force multipliers when given the gift of time through fellowships and are able to introduce innovative approaches to their areas of work disseminating their expertise to colleagues^8^. Fellows developing digital expertise provide leadership in system level understanding of digital transformation, crucial to supporting wider adoption of technology within healthcare systems^9^. With the NHS workforce facing increased staff departures and struggling to meet growing service demands^10,11^, fellowships offer healthcare professionals pathways options in portfolio careers and strategic leadership roles. By providing diversified experiences, skill development, as well as opportunities to contribute to digital transformation within the NHS, fellowships can alleviate staff burnout and enhance job satisfaction through engagement, ultimately benefiting workforce retention^12^.

### Digital fellowships

Digital Fellowships are designed to develop a range of competencies for driving digital transformation within healthcare. Central to this is the promotion digital leadership and interdisciplinary collaboration. Other key skills are an understanding of user-centred design to ensure technological implementations align with the needs of both clinicians and patients. Further, these fellowships aim to equip participants with the skills to translate clinical needs into digital realisations. They also provide insights into technology procurement strategies (identifying both upfront and hidden costs in digital solutions) and lay a solid groundwork for understanding contemporary healthcare technologies such as AI, electronic health records, wearable devices, and telemedicine.

#### Topol digital fellowship

The first and largest of these UK-based fellowships is the Topol Digital Fellowship^13^. It was created in line with the Topol Review^14^, an independent report commissioned by the NHS to explore how technological advancements and digital innovations can be integrated into the healthcare system to benefit patients and the workforce. The fellowship is open to clinical and non-clinical staff in England. This is a 12-month programme^13^, in which fellows to focus on a digital change project in their workplace. To assist in the completion of the fellow’s project, the Fellowship contains components such as: an online course outlining the basics of Machine Learning and the use of Jupyter notebooks to explore this, a programme of half-day online workshops, lunch-and-learn events, several masterclass events looking at digital innovations and their implementation, initial and concluding networking days, as well as ongoing support and mentoring throughout the fellowship. The programme has a focus on people-centred development and the program’s workshops emphasise understanding the various elements in a full project lifecycle.

**Table:** 

***Developing a digital competency framework for mental health staff***^15^
*This project revealed a need to improve digital competency among mental health staff. The result was the creation of a competency framework for digital skills in mental health, underpinned by thorough needs analysis and staff feedback. The framework, now part of a broader Health Education England digital portal, provides essential links to training resources and self-assessment tools. The outcome is an empowered workforce, capable of using digital tools without widening health inequalities, thus enhancing care for patients with severe mental illnesses*.

#### Fellowship in clinical AI

The Fellowship in Clinical AI^16^ is a 12-month programme for clinical leaders, aiming to equip them with experience in the deployment of Artificial Intelligence in healthcare (the first such systematic route in the UK). This fellowship is cited in the NHS Long Term Workforce Plan^3^ as a key approach in “upskilling and training staff to maximise technologies”. The fellowship is undertaken alongside clinical work and prioritises practical experience by attaching fellows to AI projects in NHS Trusts, embedded in a multidisciplinary team. Fellows learn to safely deploy, maintain, and evaluate AI software in real clinical workflows under the supervision and mentorship of experts in clinical AI. The fellowship’s curriculum is aligned to themes underpinning clinical AI including *AI Fundamentals*, *Regulation & Standards, Validation & Evaluation*, *Integration & Systems Impact*, and *Strategy & Culture*. This is delivered through a bespoke programme of interactive workshops throughout the year, co-developed with leaders from academia, NHS, industry, and regulators. Additional content is delivered through self-paced remote learning including King’s College London’s Innovation Scholars Programme^17^.

**Table:** 

***AI Triage in Emergency Radiology Reporting***^18^
*Radiologists can have significant backlogs in reporting non-contrast CT head scans for patients in the emergency department. This can lead to failure to prioritise reports for abnormal images of patients with time-critical abnormalities. This fellowship project focused on evaluating an industry AI algorithm for triaging CT head scans to identify abnormalities, in a multi-site deployment. The evaluation focused on measuring if there was a change in the turnaround time of reports for scans with abnormalities requiring clinical prioritisation*.

#### Florence nightingale digital fellowship/scholarships

These Scholarship and Fellowship programmes aim to cultivate digital nurse and midwifery leaders to deliver digital change and aspire to take up system leadership roles within healthcare^19^. It is structured as a 12-month secondment with the NHS England team, with an allocated mentor, various development programmes, and opportunities to work closely with the Chief Nursing Informatics Officers (CNIOs) in trusts as well as and Regional Chief Nurses and Midwives. This structure aims to equip fellows with essential skills for digital transformation, particularly providing opportunities to improve in “Digital Governance and Leadership” through personal presence and impact training as well as a leadership development project with The Kings Fund and other opportunities through a personal Continuous Professional Development (CPD) budget provided.

**Table:** 

***Improving Digital Nursing Documentation***^20^
*This project focussed on using a platform to improve digital nursing documentation at The Christie in Manchester. It resulted in staff being able to work virtually during the pandemic. Additionally, the fellowship allowed the creation of virtual placements for nursing training during the pandemic. As a result of this Fellowship, the projects completed within it were shortlisted for the Nursing Times awards*.

### Recommendations for future development

The measurement of impact generated from fellowships is challenging^21^ but vital for current and future fellowships to allow the establishment of connections between activities and outcomes. This ensure that activities align with local and national requirements as well as funding body aims.

Fellowship programmes, while serving a vital role in preparing the NHS workforce for digital transformation, are one of many tools needed. To ensure digital literacy in the wider workforce, curricula from hospitals, universities and professional bodies must evolve accordingly. Collaboration of education providers with fellowship programmes on the design and implementation of digital curricula is a golden opportunity to disseminate information from the cutting edge and share expertise. The Phillips Ives Review^22^ is a yearlong study (building upon the Topol Review’s findings) to determine the needs of the nursing and midwifery workforce to deliver healthcare in a digital age for the next 20 years. NHS Digital Academy has created a Digital competency framework for Allied Health Professionals, pharmacy workforce, and psychological practitioners^23^ which is intended to be a developmental and supportive tool to enable all these staff at different levels to meet their digital potential. As these frameworks^5^ become adopted it will cause a re-evaluation of the content of digital fellowships as digital literacy propagates through the workforce, since by design fellowships are intended to provide training *beyond* the standard curricula.

With NHS workforce planning at a critical juncture^24^ and the need to address the deficit in digital transformation capabilities^3^, the suitability of the current centrally funded model of fellowships should be questioned. Integrated Care Systems (ICSs) have a role in developing the digital workforce, addressing inequalities, and enhancing productivity and value for money^25^. One strategy for ICSs is to fund local digital fellowships, leveraging existing curricula or frameworks, and collaborating with industry for innovative solutions. The NHS Digital Academy’s Health Innovation Placement^26^ exemplifies this approach, providing exposure to start-ups/enterprises for developing technological solutions to NHS problems.

## Conclusion

Digital fellowships may serve as one of several bridges to address the digital skills gap in healthcare, aligning with the Topol Review’s goal^14^ of creating a digitally capable NHS workforce. Expansion of such opportunities globally can drive digital innovation in healthcare, benefiting academic institutions, industry, and shaping future healthcare systems. However, it’s important to acknowledge that fellowships alone cannot fully meet the digital needs of the healthcare workforce^3,14^. We call for increased collaboration among universities, industry, and professional bodies to integrate lessons from digital fellowships into relevant curricula. The educational frameworks developed by fellowship programmes can also serve as valuable resources for local models of educational delivery.
